# Hypoplastic Left Heart Syndrome Cardiomyocytes Exhibit Intrinsic Stress Vulnerabilities and Augmented Stress Responses in vitro

**DOI:** 10.1007/s12015-026-11127-3

**Published:** 2026-05-06

**Authors:** Margarida Varela, Minna Ampuja, Martin Broberg, Amanda Ramste, Virpi Talman, Emmi Helle

**Affiliations:** 1 Drug Research Program, Division of Pharmacology and Pharmacotherapy, Faculty of Pharmacy, University of Helsinki, Helsinki, Finland; 2 Stem Cells and Metabolism Research Program (STEMM), Research Programs Unit, Faculty of Medicine, University of Helsinki, Haartmaninkatu 8, Helsinki, 00290 Finland; 3 Institute for Molecular Medicine Finland (FIMM), HiLIFE, University of Helsinki, Helsinki, Finland; 4 Research Centre for Integrative Physiology and Pharmacology, Institute of Biomedicine, Faculty of Medicine, University of Turku, Kiinamyllynkatu 10, Turku, 20520 Finland; 5 New Children’s Hospital, Paediatric Research Centre, Helsinki University Hospital, Helsinki, Finland

## Abstract

**Background:**

Hypoplastic left heart syndrome (HLHS) is a severe congenital heart defect characterised by underdevelopment of left-sided cardiac structures. While genetic predisposition contributes to HLHS, the relevance of environmental stressors is increasingly recognised, yet the cellular mechanisms linking genetic susceptibility to environmental vulnerability remain unclear. We aimed to identify molecular and functional differences between cardiomyocytes derived from HLHS patients and healthy controls to uncover potential susceptibilities contributing to the HLHS phenotype.

**Methods:**

Human induced pluripotent stem cell–derived cardiomyocytes (hiPSC-CMs) from HLHS patients and healthy controls were used to examine intrinsic cellular differences. Single-cell RNA sequencing compared baseline transcriptional profiles. Functional assays assessed responses to endothelin-1 (ET-1)–induced stress, cyclic mechanical stretch, and basal or mitogen-stimulated proliferation. These approaches were used to identify intrinsic functional impairments and altered stress responses in HLHS cardiomyocytes.

**Results:**

Single-cell transcriptomics revealed downregulation of gene networks associated with cardiac stress responses, metabolic resilience, and rhythm regulation in HLHS cardiomyocytes. Regulon analysis revealed broad reductions in transcription factor activity across key cardiac regulatory networks. Functionally, HLHS cardiomyocytes showed heightened vulnerability to ET-1, with exaggerated proBNP induction compared with controls. No significant differences were observed following cyclic mechanical stretch. Basal proliferation varied across HLHS lines, while mitogen-induced proliferation remained comparable to controls.

**Conclusions:**

These findings support a model in which intrinsic molecular and functional vulnerabilities in HLHS cardiomyocytes might reduce resilience to developmental stressors. Such gene–environment interactions may contribute to HLHS pathogenesis, underscoring the interplay between genetic predisposition and environmental influences in congenital heart disease.

**Supplementary Information:**

The online version contains supplementary material available at 10.1007/s12015-026-11127-3.

## Background

Congenital heart diseases (CHDs) are structural malformations of the cardio-circulatory system that arise during embryonic development. Encompassing a broad spectrum of subtypes, CHDs range from relatively common minor lesions to complex and life-threatening anomalies such as hypoplastic left heart syndrome (HLHS). HLHS represents one of the most severe forms of CHD, characterised by the profound underdevelopment of left-sided cardiac structures, including the aortic and mitral valves, the left ventricle, and the aorta [1]. This defect critically impairs the ability of the left ventricle to support systemic circulation, rendering the condition fatal without surgical intervention [2, 3].

The aetiology of CHDs is complex, and the precise pathogenic mechanisms underlying these disorders remain incompletely understood. Both genetic predisposition and environmental stressors contribute to the defects. In the context of HLHS, substantial efforts have been devoted to elucidating its molecular and genetic underpinnings. While HLHS occasionally co-occurs with syndromic disorders [4–6], it predominantly arises as an isolated defect. Genetic investigations have implicated a range of critical genes, including transcription factors (TF) such as *NKX2-5* [7–9], *TBX5* [10], *HAND1* [11, 12], as well as receptor and structural protein genes such as *NOTCH1* [13–16] and *MYH6* [17, 18]. These findings underscore the diversity of genetic disruptions capable of derailing cardiac development. Nevertheless, genetic susceptibility alone does not fully account for the phenotypic heterogeneity and variable phenotype, suggesting that additional factors contribute significantly to disease pathogenesis.

Increasing attention has been directed towards the role of environmental influences, which may act synergistically with genetic defects [19]. Maternal diabetes and obesity [20–25], hyperglycaemia [24, 26–28], oxidative stress [22, 23], and aberrant haemodynamic forces in the developing foetus [29–33] have emerged as pivotal external factors capable of disrupting foetal cardiac development. These perturbations, often mediated through alterations at the maternal-foetal interface, may exert profound effects during critical windows of gestation.

The advent of human induced pluripotent stem cells (hiPSCs) [34, 35] has ushered in a new paradigm in cardiovascular disease modelling. hiPSCs, reprogrammed from patient-specific cells, retain the full complement of the individual’s genetic information. This technology enables the generation of cardiomyocytes (CMs) that faithfully recapitulate patient-specific genetic backgrounds, providing an unprecedented platform for dissecting the cellular and molecular mechanisms underlying congenital heart defects such as HLHS.

In this study, we examined both healthy and HLHS-derived cardiomyocytes to investigate their inherent differences as well as their capacity to respond to environmental cues. Acknowledging that heart development results from a dynamic interplay between genetic programming and extrinsic signals, we sought to model key developmental stressors in vitro. We performed single-cell RNA sequencing (scRNA-seq) to profile transcriptomic changes and identify disease-associated molecular signatures. Following this, we applied three distinct stimuli to model critical aspects of cardiac development: Endothelin-1 (ET-1), a known pro-hypertrophic hormone elevated in adverse maternal environments, was used to assess hypertrophic responses. Cyclic mechanical stretch simulated the biomechanical forces experienced by the foetal heart, and pro-proliferative compounds were used to evaluate the cells’ proliferative potential. These stimuli were chosen to represent essential developmental processes relevant to both normal cardiac formation and the pathogenesis of HLHS. Together, these complementary approaches allowed us to interrogate intrinsic deficits and impaired adaptability that may contribute to the abnormal cardiac morphogenesis characteristic of HLHS.

## Materials and Methods

### hiPSC Lines and Culture

Eight hiPSC lines were used in this study, including four from healthy controls (HEL24.3 [36], HEL47.2 [37], HEL46.11 [38], and K1), three from individuals with HLHS (HEL149.2, HEL218.6, HEL169.4), and one from an individual with left ventricular outflow tract obstruction (LVOTO; HEL216.6). All lines, except for K1, which was was kindly gifted by Anu-Suomalainen-Wartiovaara, were obtained from Biomedicum Stem Cell Center Core Facility. To improve readability throughout this study, these lines are hereafter referred to as follows: HEL24.3 as Healthy 1, HEL47.2 as Healthy 2, HEL46.11 as Healthy 3, K1 as Healthy 4, HEL149.2 as HLHS 1, HEL218.6 as HLHS 2, HEL169.4 as HLHS 3, and HEL216.6 as HLHS 4. Detailed information on donor sex, genetic variants, and cardiac phenotypes is provided in (Additional file 1: Table S1). The cell lines were created using retroviral or Sendai virus-mediated transduction with Yamanaka reprogramming factors *OCT3/4*, *SOX2*, *KLF4*, and *MYC* [34, 35] as previously described by Trokovic et al. 2015 [36, 37]. All hiPSC lines used in this study were evaluated for pluripotency and regularly tested for genomic stability through karyotyping. The hiPSCs were seeded on plates thin-coated with Matrigel™ (Corning, #354277; diluted at 1:200) and cultured in Essential 8™ medium (Thermo Fisher Scientific, #A1517001). Cells were passaged twice weekly using PBS with 0.5 mM EDTA until at least passage 20 before differentiation. The absence of mycoplasma contamination was routinely confirmed using the MycoAlert Mycoplasma Detection Kit (Lonza, #LT07-218).

### Differentiation of hiPSCs into CMs

Cardiomyocyte (CM) differentiation was induced in a monolayer culture as previously described by Helle et al. (2021) [39]. Briefly, hiPSCs were seeded at a 1:10–1:15 ratio on 12-well plates coated with Matrigel™ (Corning, #354277). Upon reaching 80–90% confluency (Day 0), cardiac mesoderm induction was initiated by replacing Essential 8™ with RPMI 1640 medium containing L-glutamine and glucose (Corning, #10–040-CV), supplemented with B-27™ Supplement minus insulin (Thermo Fisher Scientific, #A1895601) and 4–5 µM GSK-3α/β inhibitor CHIR-99021 (CHIR, Selleck Chemicals, #S2924). On Day 1, fresh medium containing CHIR was added. After 48 h (Day 2), the culture medium was replaced with fresh RPMI 1640, replacing CHIR with 5 µM IWR-1 (Sigma-Aldrich, #I0161) and replenished on Day 3. From Day 4 to Day 5, cells were maintained in fresh RPMI 1640 supplemented with B-27™ minus insulin. On Day 6 and Day 7, the medium was changed to RPMI 1640 with B-27™ containing insulin (Thermo Fisher Scientific, #17504044). Feeding on Day 8 depended on the appearance of the cells: if significant cell death was observed, feeding was performed to help reduce the dead cells. From Day 9 to Day 11, to enrich for cardiomyocytes, the medium was replaced with RPMI 1640 without glucose (Thermo Fisher Scientific, #11560406), supplemented with B-27™ containing insulin and 5 mM sodium L-lactate (Sigma-Aldrich, #71718). No medium change was performed on Day 12. On Day 13, robust beating of the monolayer was typically observed, and the differentiated hiPSC-CMs were passaged. Cells were detached using Accutase (0.4 mL for 12-well plates, 0.6 mL for 6-well plates), quenched with RPMI 1640 culture medium with L-glutamine with glucose supplemented with B-27™ (Thermo Fisher Scientific, #17504044), and centrifuged for 4 min at 200 × g. Cells were resuspended in the same medium with 10 µM Rock inhibitor (StemCell Technologies, #Y-27632) and replated onto a new plate. From the following day, cells were maintained in RPMI without glucose, supplemented with B-27 containing insulin and 5 mM sodium L-lactate and fed every other day until day 25–30, when they were moved to RPMI with glucose, supplemented with B-27 containing insulin until they were used in experiments. All experiments were conducted within the early maturation phase of hiPSC-derived cardiomyocytes (approximately Days 20–40 of differentiation).

To ensure experimental consistency, only differentiations achieving a cardiomyocyte purity of ≥ 80%, as determined by cardiac troponin T immunostaining, were included in this study. All functional assays in this study (cell proliferation, endothelin-1 stimulation, and cyclic mechanical stretch) were performed on a predefined subset of hiPSC-derived cardiomyocyte lines, comprising two independent healthy control lines (Healthy 1 and Healthy 2) and two independent HLHS lines (HLHS 1 and HLHS 2), randomly selected prior to experimentation. In contrast, single-cell RNA sequencing analyses were performed using four healthy control lines and four HLHS lines.

### CM Processing for scRNA-seq

hiPSC-CMs were harvested for scRNA-seq at differentiation day 38 or 39, with the exception of HEL47.2, which was collected at day 27. Following established protocols [39, 40], the cells were counted and washed with 0.04% BSA in PBS. For scRNA-seq preparation, patient-derived and control-derived cells were pooled separately by combining equal contributions from each respective cell line. The pooled samples were then sent to the Institute for Molecular Medicine Finland (FIMM) for processing and sequencing using the 10x Genomics Single Cell Protocol.

### scRNA-seq Bioinformatics

In the scRNA-seq analysis, four HLHS lines and four healthy control lines were used. The healthy control lines were pooled and analysed as a single control sample. To distinguish the sequencing data corresponding to each HLHS line within the pooled sample, we employed FreeBayes v1.3.1 [41] to call genetic variants from the exome data of the four HLHS individuals. These variant calls were subsequently used as input for Demuxlet [42], enabling the assignment of individual cells to their respective cell lines within the combined single-cell RNA-sequencing dataset. Downstream analysis was performed in R (2024.09.1 + 394) using the Seurat (version 5.1.0) package. A total of 17,980 high-quality cells were isolated to identify distinct cell populations and enable subsequent downstream analyses. The following quality control criteria were applied to the data: [1] Genes expressed in fewer than 200 cells or in more than 8,000 cells were excluded; [2] Cells with fewer than 200 or more than 8,000 detected genes were removed, as these may indicate low-complexity cells or doublets; [3] Cells exhibiting more than 30% mitochondrial gene expression were excluded to eliminate potentially damaged or stressed cells. Data normalization was performed using the *“NormaliseData”* function in Seurat, and the top 2,000 most variable genes were identified with the *“vst”* method via the *“FindVariableFeatures”* function. Integration anchors were computed using *“FindIntegrationAnchors”* to align shared features across datasets, and the data were integrated using *“IntegrateData”*. These genes were subsequently analysed using principal component analysis (PCA) for linear dimensionality reduction.

### Cell Type Identification and Cluster Analysis

Following data integration and normalization, we performed unsupervised clustering to identify distinct cellular populations within the cardiac dataset and assess compositional differences between HLHS and healthy samples. The two-dimensional Uniform Manifold Approximation and Projection (UMAP) was performed using the RunUMAP function in Seurat on the first 30 principal components. Graph-based clustering was applied to identify cell populations based on gene expression profiles using the FindClusters function, and a resolution of 0.4 was selected for downstream analysis, resulting in 14 distinct clusters. The resulting UMAP projection was used to visualize the clusters, and cell types were annotated based on known marker genes. Cluster-specific markers were identified using the FindAllMarkers function and annotated using published literature.

Cell populations were classified as follows: early-state cardiomyocytes (clusters 0, 4, 5, 6, 7, 8, 10, 12, 13) expressing cardiac transcription factors (*NKX2-5*, *TBX5*, *GATA4*, *ISL1*) and sarcomeric genes; later-state cardiomyocytes (clusters 1, 2, 3, 11) with robust expression of structural genes (*MYH6*, *MYL7*, *TNNT2*, *TTN*); and cardiac fibroblasts/smooth muscle cells (cluster 9) expressing *POSTN*, *COL1A1*, *TAGLN*, and *ACTA2*. This classification reflects relative differentiation states inferred from transcriptomic profiles rather than fully mature cardiomyocyte phenotypes.

To assess differences in cluster composition between healthy and HLHS samples, the proportion of cells in each cluster was calculated relative to the total number of cells per sample or group. Statistical significance was evaluated using chi-square tests with Benjamini-Hochberg FDR correction. Comparisons were performed between Healthy versus all HLHS samples combined, and between Healthy versus each individual HLHS line separately.

### Differential Gene Expression Analysis

Differential gene expression analysis was performed on cardiomyocyte populations (clusters 0–8, 10–13) using the FindMarkers function in Seurat with the default statistical test, Wilcoxon rank-sum test. Two analytical approaches were employed: [1] overall comparison of Healthy versus all HLHS samples combined, and [2] line-specific comparisons of Healthy versus each individual HLHS line (HLHS 1–4). Differentially expressed genes (DEGs) were selected only if they met the following criteria: average log₂ fold change > 0.5 or < −0.5 and adjusted p-value < 0.05. To identify core transcriptional changes, we examined genes consistently dysregulated in the same direction across multiple HLHS samples.

For visualization purposes in volcano plots, genes with adjusted p-values of 0 (*S100A10*, *NPPB*, *NPPA*,* RPS27*, *RPS29*, *RPS26*) were assigned an adjusted p-value of 1⁻³²⁰ to enable numerical calculation of -log₁₀(adjusted p-value). Key differentially expressed genes were visualized using volcano plots and heatmaps. Gene ontology enrichment analysis was performed to identify biological processes associated with differentially expressed genes, with results displayed as bubble plots showing fold enrichment and statistical significance.

### Gene Ontology Enrichment Analysis of Differentially Expressed Genes

The set of differentially expressed genes identified across all four HLHS-CM lines was used as input for Gene Ontology (GO) enrichment analysis. This was conducted using the Functional Annotation Tool within the Database for Annotation, Visualization and Integrated Discovery (DAVID) and the DAVID Knowledgebase (v2023q4, updated quarterly; https://davidbioinformatics.nih.gov/tools.jsp) [43, 44]. The gene list was uploaded using official gene symbols as identifiers, and Homo sapiens was selected as the background species. From the resulting Annotation Summary, the Gene Ontology Biological Processes category was selected for further analysis. GO terms with a false discovery rate (FDR)-adjusted p-value < 0.05 were considered significantly overrepresented.

### Transcription Factor Regulon Analysis

To systematically evaluate transcriptional regulatory changes in HLHS, we performed regulon activity analysis across all cardiac cell populations. Transcription factors were identified from the Lambert et al. (2018) validated human TF database [45], filtering for those expressed in ≥ 5% of cells. For each TF, target genes were predicted by calculating Spearman correlation coefficients (threshold > 0.1), retaining only regulons with ≥ 10 target genes.

Regulon activity in individual cells was quantified using AUCell (v1.24.0), with the AUC maximum rank parameter set to 5% of total genes. Differential regulon activity between HLHS and healthy samples was assessed using Wilcoxon rank-sum tests with Benjamini-Hochberg FDR correction (FDR < 0.05). For each regulon, we calculated log₂ fold change and percent change. Cardiac-specific TF regulons were identified by cross-referencing TFs with cardiac development Gene Ontology terms.

For the 90 cardiac TF regulons, GO enrichment analysis was performed on target genes using clusterProfiler (v4.10.0) with FDR correction (FDR < 0.05). For each regulon, the top 3 most significantly enriched pathways were retained and manually grouped into four functional categories for downstream interpretation.

### Cell Proliferation Assay

To induce and assess cell proliferation, hiPSC-CMs at Days 20 and 33 of differentiation were selected to capture the transition from higher to reduced proliferative activity during early maturation. Day 20 represents an early stage shortly after cardiomyocyte differentiation, when hiPSC-derived cardiomyocytes retain substantial proliferative capacity. By contrast, at Day 33, proliferative activity remains detectable but is markedly reduced, enabling assessment of the progressive decline in cardiomyocyte cell cycle activity, as previously described by Kodo et al. [46].

Cells were treated with a combination of CHIR99021 and SB203580, which work synergistically to promote cardiomyocyte proliferation [47]. CHIR99021, a GSK-3 inhibitor, activates Wnt/β-catenin signalling [48], while SB203580 inhibits p38 MAPK-mediated cell cycle suppression [49], together producing more robust mitogenic effects than either compound alone. hiPSC-CMs were seeded onto Matrigel-coated 96-well PhenoPlates (PerkinElmer, #6055300) at a density of 4.5 × 10^4^ cells per well and allowed to adhere overnight. The hiPSC-CMs were then divided into two treatment groups: the experimental group was treated with a combination of 5 µM CHIR99021 (CHIR, Selleck Chemicals, #S2924) and 10 µM SB203580 (SB, Selleck Chemicals, #S1076) alongside 10 µM Bromodeoxyuridine (BrdU, Abcam, #ab142567) for 24 h, while the control group received vehicle (DMSO) with BrdU for the same period.

### Induction of Cardiomyocyte Hypertrophy

Hypertrophic stress experiments were performed at later stages of differentiation to capture a more advanced maturation state within the early differentiation window. At this stage (Days 30–40), hiPSC-derived cardiomyocytes have progressed along the maturation trajectory and display more defined structural and functional properties, enabling consistent and reproducible transcriptional and phenotypic responses to hypertrophic and mechanical stimuli, as reported by Pohjolainen et al. [50, 51].

#### Endothelin-1 (ET-1) treatment

To induce cell hypertrophy, Day 33 hiPSC-CMs were seeded onto 96-well Matrigel-coated PhenoPlates (PerkinElmer, #6055300) at 4.5 × 10^4^ cells per well and allowed to attach overnight. The cells were then exposed to either ET-1 or a vehicle control consisting of 1% bovine serum albumin (BSA; Sigma-Aldrich, #A9418) in Dulbecco’s Modified Eagle Medium (DMEM; Sigma-Aldrich D-7777) for 24 h. Brefeldin A (1000X Solution; Invitrogen, #B7450) was added to the last 3 h to inhibit the exocytosis of pro-B-type natriuretic peptide (proBNP)-containing vesicles as described previously [52].

#### Cyclic mechanical stretch

To assess the effect of cyclic mechanical loading on the expression of hypertrophy-related genes, hiPSC-CMs aged between Days 30–40 were cultured on BioFlex^®^ plates at a cell density of 8.5 × 10^5^ per well. These cells were then subjected to 24–48 h of cyclic mechanical strain using an FX-5000 Tension System (Flexcell International Corporation). Equibiaxial cyclic stretch was applied in two-second cycles (0.5 Hz) at a level sufficient to promote cyclic 10 to 21% elongation, corresponding to 42–80 kPa at the point of maximal distension of the culture surface [51]. Unstretched control cells from the same differentiation were maintained in BioFlex^®^ plates in the same environmental conditions, but no stretch was applied.

### RNA extraction, cDNA Synthesis and qPCR

For qPCR, total RNA was purified using the NucleoSpin RNA Kit (Macherey-Nagel, #740961) according to the manufacturer’s protocol. The cells were lysed in 350 μl of RA1 lysis buffer supplemented with 1% β-mercaptoethanol and stored at − 80 °C until RNA isolation. Analysis of the RNA concentration and quality was performed with a NanoDrop 1000 spectrophotometer (Thermo Fisher Scientific). Total RNA (100–500 ng) was reverse transcribed in 10 μl reactions by using the Transcriptor First Strand cDNA Synthesis Kit (Roche, #04897030001) using random hexamer primers and an MJ Mini Personal Thermal Cycler (Bio-Rad). The cDNA was diluted 1:10 in PCR grade H_2_O and stored at − 20 °C. Commercial TaqMan^®^ Gene Expression Assays (Thermo Fisher Scientific), detailed in Additional file 1:Table S2, were used in conjunction with the LightCycler 480^®^ Probes Master reagent (Roche) following the manufacturer’s protocols.Gene expression was analysed using the LightCycler^®^ 480 Real-Time PCR System (Roche) with 4.5 μl of cDNA in a 10 μl reactionvolume on a white LightCycler 480^®^ Multiwell Plate 384 (Roche). No-template controls were included to confirm the absence of PCR contamination. Each reaction was performed in triplicate, with the mean of technical replicates representing a single biological replicate (*n*= 1). Outliers within technical replicates were identified using Grubbs’ test at a significance level of 0.05 and subsequently excluded fromthe analysis. Relative gene expression levels were quantified using the 2 − ΔΔCt method, referenced to the average of *ACTB* and *18S* rRNA housekeeping genes and normalised to the average of untreated biological replicates of Healthy 2.

### Immunofluorescence Staining

For immunofluorescence staining, all procedures were carried out at room temperature (RT) unless otherwise specified. hiPSC-CMs were washed twice with phosphate-buffered saline (PBS) and fixed with 4% paraformaldehyde for 15 min. Cells were then washed 3 × 5 min with PBS. Permeabilization was performed using 0.1% Triton X-100 (AppliChem, #A4975) in PBS for 10 min, followed by 2 × 5 min washes with PBS. For BrdU staining, DNA was hydrolysed with 2 M hydrochloric acid for 30 min, neutralised with 0.1 M sodium borate (pH 8.5) for 30 min, and washed 3 × 5 min with PBS. To prevent nonspecific binding, the cells were blocked with 4% foetal bovine serum (FBS) (Thermo Fisher Scientific, #10500064) in PBS for 45 min. The cells were then incubated for 60 min with the following primary antibodies diluted in 4% FBS in PBS: cardiac troponin T (cTnT) antibody (Abcam, #ab45932, 1:800), BrdU antibody (Abcam, #ab6326, 1:250), or proBNP antibody (Abcam, #ab13115, 1:250), followed by 3 × 5 min washes with PBS. For validation of selected transcriptomic targets, cells were incubated overnight at 4 °C with ENO1 antibody (Antibodies.com, #A85406, 1:3000) and MYH6 antibody conjugated to CoraLite^®^ Plus 488 (Proteintech, #CL488-22281, 1:200). Where required, cells were subsequently incubated for 45 min with Alexa Fluor^®^-conjugated secondary antibodies: Alexa Fluor™ 488 (Invitrogen, #A-11029, 1:200), Alexa Fluor™ 546 (Invitrogen, #A-11035, 1:200), Alexa Fluor™ 647 (Invitrogen, #A-21236 and #A-21247, 1:200), together with 4′,6-diamidino-2-phenylindole (DAPI) (Sigma-Aldrich, #D9542, 1 µg/mL) for nuclear staining, followed by 3 × 5 min washes with PBS. Finally, cells were stored in PBS at 4 °C until imaging.

### Imaging and Analysis

Automated fluorescence microscopy was performed using the ImageXpress Micro Confocal imaging system (Molecular Devices). Representative images were acquired with a Nikon 20× Plan Apo 0.5 NA air objective, while images for downstream analysis were captured using a Nikon 10× Plan Apo objective. The acquired images were analysed using MetaXpress software (Molecular Devices). First, the nuclei were identified based on DAPI staining, and cardiomyocytes were distinguished by the presence of cTnT staining in the cytoplasm, with non-myocytes excluded based on the absence of cTnT. To identify BrdU-positive CMs, nuclei confirmed as CMs by DAPI and cTnT staining were cross-referenced with BrdU staining, and the overlap of these signals was defined as BrdU-positive CMs. The threshold for BrdU positivity was manually adjusted in each experiment to account for variations in staining intensity. For proBNP quantification, the average intensity of proBNP staining was measured within the perinuclear region, defined as a 10-pixel ring surrounding each CM nucleus. Similarly to the BrdU analysis, cells were categorized as proBNP-positive or proBNP-negative based on staining intensity, with thresholds manually adjusted for each experiment to account for staining variations. For validation of selected transcriptomic targets, average fluorescence intensities of ENO1 and MYH6 were quantified in cardiomyocytes identified by cTnT staining. Nuclear regions were defined based on DAPI staining. ENO1 fluorescence intensity was measured both within the nuclear mask and across the entire cardiomyocyte area to assess potential differences in subcellular localisation and overall protein abundance. Whole-cell ENO1 intensity was quantified within the cytoplasmic area defined by cTnT staining. MYH6 fluorescence intensity was measured across the entire cardiomyocyte area. As MYH6 is itself a sarcomeric cardiomyocyte marker, its staining largely overlapped with cTnT signal, confirming cardiomyocyte identity.

### Data Analysis

Statistical analyses were carried out using GraphPad Prism 8 software. Data are presented as mean ± standard error of the mean (SEM), unless otherwise stated. A p-value of less than 0.05 was considered statistically significant. All experiments were conducted with a minimum of three biological replicates (n), unless otherwise stated. Statistical analysis of the scRNA-seq data was performed using pairwise comparisons between each HLHS sample and pooled healthy controls using the Wilcoxon rank-sum test implemented in the “FindAllMarkers” function of the Seurat package. In addition, a combined analysis comparing all HLHS samples to pooled healthy controls was conducted using the same statistical method. Genes were considered differentially expressed if they exhibited an absolute log₂ fold change greater than 0.5 and a Bonferroni-adjusted p-value of less than 0.05. The Bonferroni correction was applied to adjust for multiple testing across all genes in the dataset. Statistical analysis of protein-level validation experiments was performed using Brown–Forsythe and Welch ANOVA followed by Dunnett’s T3 multiple-comparisons test to account for unequal variances. Statistical significance for functional assays was determined using two-way ANOVA followed by Tukey’s post hoc multiple-comparisons test, with fold change in proliferation calculated for each biological replicate as the ratio of treated to corresponding control values.

## Results

### Identification of Cell Types

**Fig. 1:**
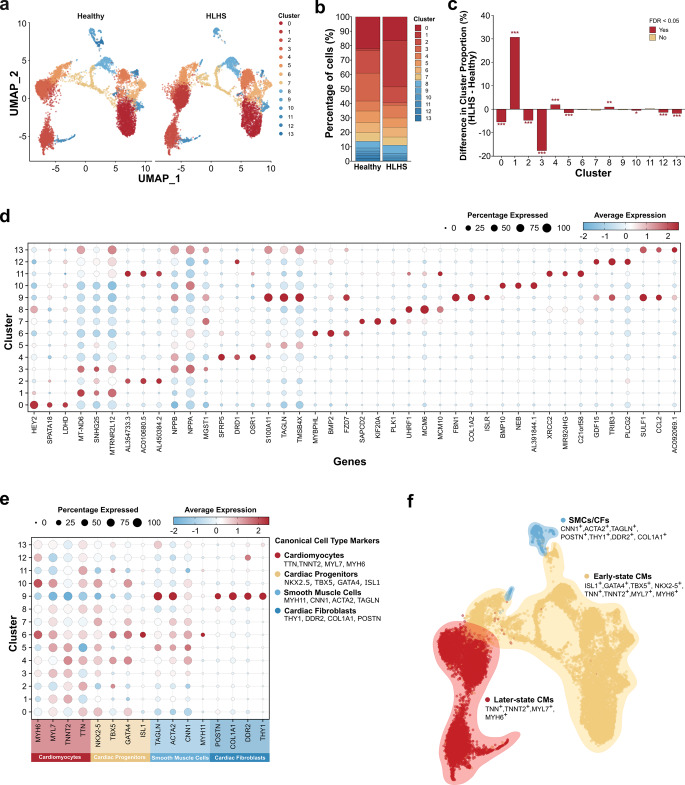
Single-cell transcriptional profiling of healthy and HLHS cardiomyocytes. Analysis compares HLHS patient lines (n=4) to a pooled healthy control (4 cell lines combined).** (a)** UMAP visualisation of integrated single-cell RNA sequencing data from healthy and HLHS samples, split by group. Cells are coloured by cluster identity (0-13) using unsupervised graph-based clustering (resolution = 0.4). Each dot represents a single cell (n = [total cells]). **(b)** Stacked bar chart showing the relative proportion of each cluster within healthy and HLHS samples. Clusters are ordered 0-13 from bottom to top, with colours corresponding to panel **(a)**. **(c)** Bar chart depicting the percentage point difference in cluster proportions between HLHS and healthy samples (HLHS - Healthy). Bars are coloured by statistical significance after FDR correction (red: FDR < 0.05; beige: not significant). Asterisks indicate significance levels: *p < 0.05, **p < 0.01, ***p < 0.001. **(d)** Dot plot showing expression of the top 3 differentially expressed marker genes for each cluster. Dot size represents the percentage of cells expressing each gene within a cluster; dot colour indicates average scaled expression level (blue: low, white: intermediate, red: high). **(e)** Dot plot showing expression of canonical cardiac cell type markers across clusters. Dot size and colour as in panel **(d)**. **(f)** UMAP visualisation showing annotated cell populations based on marker gene expression. Three major cell types were identified: Later-state CMs (red), Early-state CMs (beige/yellow), and SMCs/CFs (blue)

Unsupervised graph-based clustering of the single-cell RNA sequencing data identified 14 distinct cell clusters (clusters 0–13) across both conditions (Fig. 1a). Transcriptional profiling revealed largely overlapping distributions between healthy and HLHS cardiomyocytes, suggesting considerable similarity in overall cellular heterogeneity.

While the general clustering pattern was comparable across samples, differences in cluster distribution were observed (Fig. 1b and c) Cluster 1 showed the largest enrichment in HLHS samples, while cluster 3 exhibited the lowest number of HLHS cells. Individual HLHS line analysis is shown in (Additional file 1: Fig. S1a-c).

To characterise the molecular identity of each cluster, we identified differentially expressed marker genes, with the top 3 markers per cluster displayed in (Fig. 1d). Based on the canonical marker expression patterns, we classified the 14 clusters into three major cell populations (Fig. 1e and f). Clusters 0, 4, 5, 6, 7, 8, 10, 12, and 13 robustly expressed both cardiac-specific transcription factors (*NKX2-5*, *TBX5*, *GATA4*, and *ISL1*) and structural genes essential for cardiomyocyte function (*MYH6*, *MYL7*, *TNNT2*, and *TTN*), consistent with early-state cardiomyocytes in which developmental regulatory networks remain active. Such co-expression of developmental transcription factors together with sarcomeric genes is commonly observed in early-stage hiPSC-derived cardiomyocytes, where structural programs are already active while developmental regulatory networks remain engaged. In contrast, clusters 1, 2, 3, and 11 displayed a gene expression profile consistent with later-state cardiomyocytes along the cardiomyocyte differentiation continuum, showing reduced expression of developmental transcription factors while maintaining robust expression of functional sarcomeric components. Cluster 9 displayed a distinct expression profile, with relatively low expression of cardiac markers but high levels of fibroblast markers (*POSTN*, *COL1A1*, *DDR2*, *THY1*) and smooth muscle cell markers (*TAGLN*, *ACTA2*, *CNN1*, *MYH11*), identifying this cluster as a non-myocyte population. Pluripotency markers (*SOX2*, *NANOG*, *POU5F1*, *LIN28A*) and primitive streak markers (*FOXA2*, *TBXT*, *EOMES*, *GSC*) were undetectable across all clusters (Additional file 1: Fig. S1d), confirming the absence of undifferentiated cells or early mesodermal progenitors. Importantly, these classifications reflect relative differentiation states inferred from transcriptomic profiles and should not be interpreted as evidence of fully mature cardiomyocyte phenotypes, which require structural, metabolic, and electrophysiological characterization.

### Differential gene expression and functional enrichment analysis

**Fig. 2:**
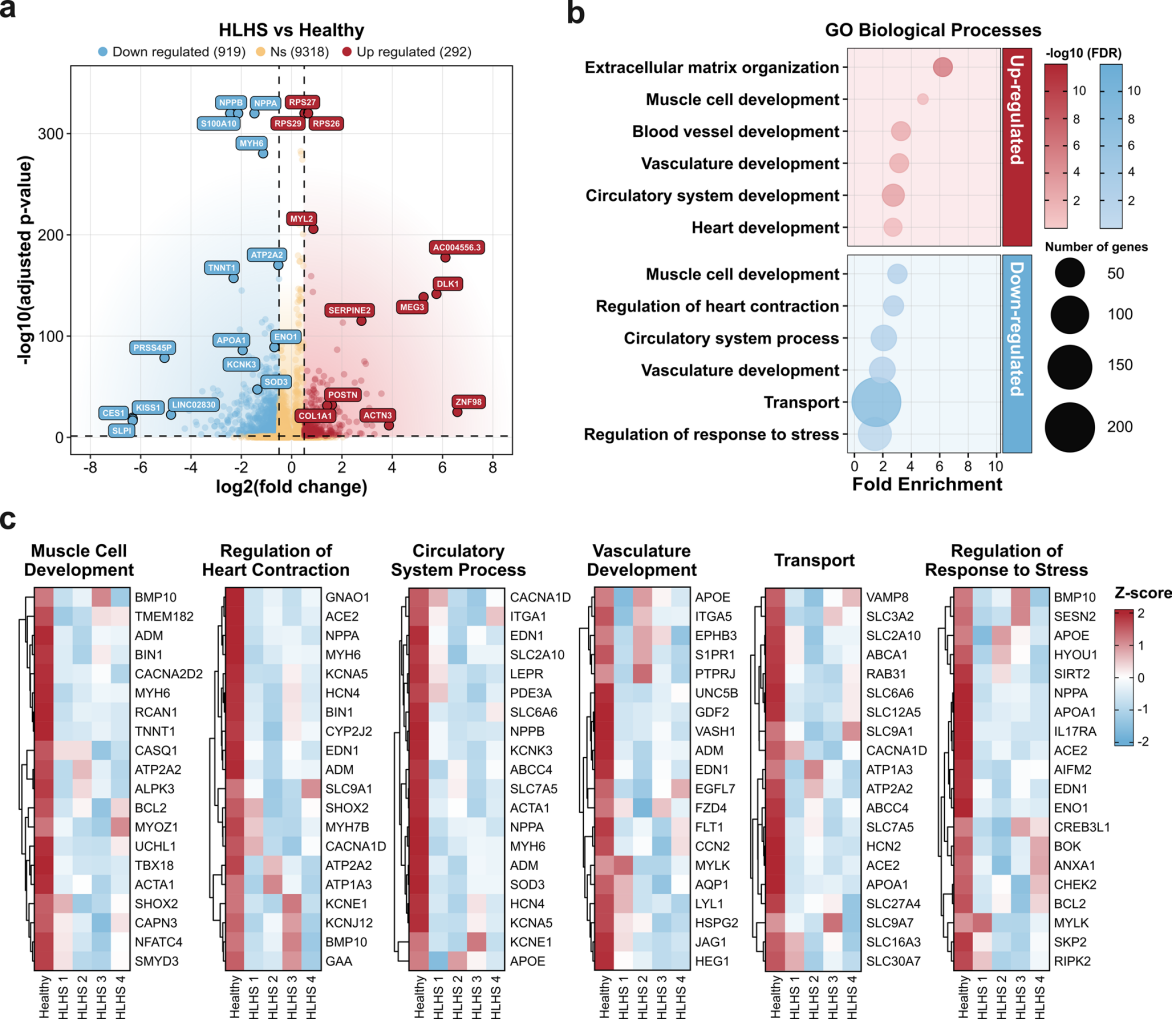
Differential gene expression and functional enrichment in HLHS. Analysis compares HLHS patient lines (*n* = 4) to a pooled healthy control (4 samples combined). **(a)** Volcano plot showing differentially expressed genes between HLHS and healthy samples. Blue dots represent downregulated genes (919), red dots represent upregulated genes (292), and yellow dots represent genes that were not differentially expressed (9,318). Selected key genes are labeled. Vertical dashed lines indicate log2(fold change) thresholds of −0.5 and 0.5; horizontal dashed line indicates adjusted p-value threshold of 0.05. **(b)** Gene Ontology (GO) Biological Processes enrichment analysis of differentially expressed genes in HLHS. Circle size represents the number of genes in each GO term (range: 9–220 genes). Colour intensity indicates statistical significance (-log10 p-value), with darker colours representing more significant enrichment. X-axis indicates fold enrichment. Top panel shows enriched processes in upregulated genes; bottom panel shows enriched processes in downregulated genes. **(c)** Heatmaps showing expression patterns of key genes within six selected enriched biological processes across individual samples. Colour scale represents log2(fold change) values from − 2 (blue, downregulated) to 2 (red, upregulated)

To identify the molecular alterations underlying the compositional differences between healthy and HLHS hiPSC-CMs, we performed differential gene expression analysis. This analysis revealed substantial transcriptomic differences, identifying 1,211 differentially expressed genes, of which 292 were upregulated and 919 downregulated in HLHS compared to healthy cardiomyocytes (Fig. 2a, Additional file 2: Table S1). Individual HLHS line analysis is shown in (Additional file 1: Fig. S2a-d).

Gene Ontology (GO) enrichment analysis of differentially expressed genes (Fig. 2b; Additional file 3: Table S1) revealed significant involvement in a range of developmental and functional pathways. Notably, many of the enriched biological processes were observed among the downregulated genes, including processes related to circulatory system, vasculature development, and muscle cell development. To explore these in more detail, we generated heatmaps of representative genes from the downregulated categories (Fig. 2c). Among the downregulated genes in circulatory system processes, key genes included *EDN1*, *SOD3*, and *ACE2*. Genes involved in vasculature development showed reduced expression, including *APOE*, *EDN1*, and *JAG1*. Muscle cell development genes such as *MYH6*, *BMP10*, and *TNNT1* were also downregulated in HLHS samples. Additional downregulated genes and their associated terms included genes involved in regulation of heart contraction (*MYH6*, *HCN2*, *HCN4*, *KCNA5*, *KCNE1*), regulation of response to stress (*ENO1*, *SESN2*, *APOA1*, *NPPA*), and transport (*SLC7A5*, *ATP2B4*). These findings indicate that HLHS hiPSC-CMs exhibit widespread downregulation of genes involved in cardiac development, contractile function, circulatory system processes, and cellular stress responses.

To experimentally assess whether selected transcriptomic changes were reflected at the protein level, we performed immunofluorescence staining for ENO1 and MYH6, two genes identified as significantly dysregulated in the single-cell RNA sequencing analysis. Quantitative analysis revealed reduced ENO1 protein levels in HLHS cardiomyocytes compared with healthy controls. Nuclear ENO1 fluorescence intensity was significantly decreased in HLHS 1 relative to pooled healthy controls, while HLHS 2 showed a similar directional reduction (Additional file 1: Fig. S3a). Whole-cell ENO1 fluorescence intensity was also significantly reduced in HLHS 2 and showed a decreasing trend in HLHS 1 (Additional file 1: Fig. S3b). For MYH6, protein quantification revealed a modest reduction in HLHS cardiomyocytes compared with healthy controls, consistent with the direction of the transcriptomic changes observed in the RNA-seq dataset (Additional file 1: Fig. S3c).

### TF Regulon Activity in HLHS Cardiomyocytes

**Fig. 3:**
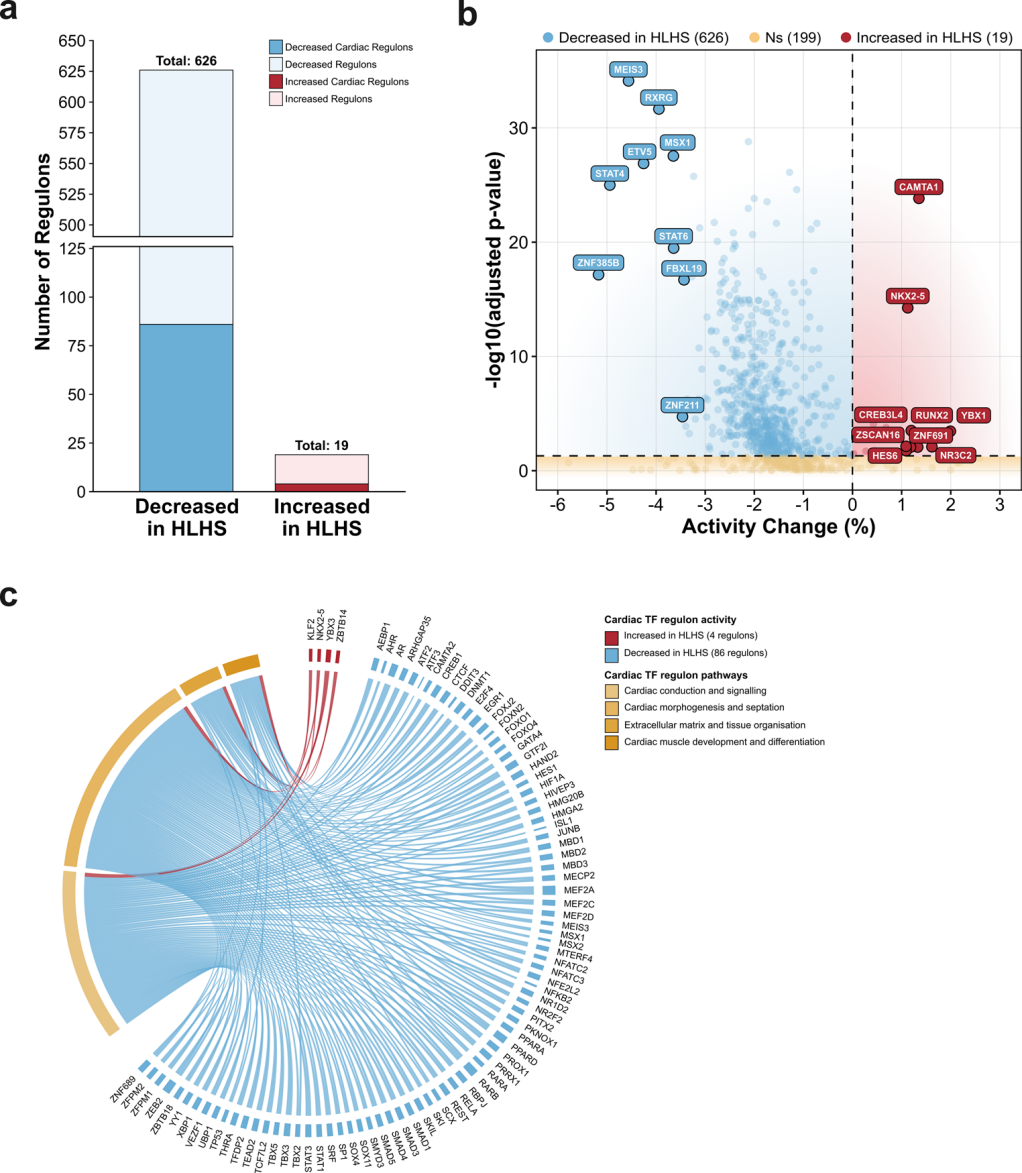
Transcription factor regulon analysis in healthy and HLHS cardiomyocytes.** (a)** Stacked bar chart comparing differential regulon activity between HLHS and healthy cardiomyocytes. Of 645 significantly differentially active regulons, 626 showed decreased activity (dark blue: 86 cardiac-specific regulons; light blue: 540 non-cardiac regulons) and 19 showed increased activity (dark red: 4 cardiac-specific regulons; light pink: 15 non-cardiac regulons) in HLHS compared to healthy controls. **(b)** Volcano plot showing activity changes across all 844 analysed regulons. X-axis represents percent change in regulon activity; y-axis shows statistical significance (-log10 adjusted p-value). Blue points indicate decreased activity in HLHS (*n* = 626 significant), red points indicate increased activity in HLHS (*n* = 19 significant), and yellow points represent non-significant regulons (*n* = 199). Horizontal dashed line marks FDR < 0.05 significance threshold; vertical dashed line indicates no change. Top 10 most changed regulons in each direction are labeled. **(c)** Circus plot showing connections between 90 cardiac-specific TF regulons (outer ring: red = increased activity in HLHS, blue = decreased activity in HLHS) and four functional pathway categories derived from GO enrichment analysis (inner segments, coloured by pathway type). Ribbon width is proportional to GO enrichment strength

To understand regulatory mechanisms underlying transcriptional changes in HLHS, we performed transcription factor regulon analysis. This approach reveals whether TFs are actively regulating their downstream targets, which can be disrupted in disease even without changes in TF expression itself. We constructed and analyzed 844 TF regulons, of which 645 showed statistically significant differential activity between HLHS and healthy samples (FDR < 0.05;). The vast majority (626 regulons, 97.1%) exhibited decreased activity in HLHS compared to healthy controls, whereas 19 regulons (2.9%) showed increased activity (Fig. 3a and b). A total of 90 of the differentially active regulons were driven by cardiac-specific transcription factors, with 86 (95.6%) showing reduced activity and 4 (4.4%) showing increased activity in HLHS (Fig. 3a). The most increased regulons included *CAMTA1*, *NKX2-5*, and *YBX1*, while the most decreased included *MEIS3*, *RXRG*, and *ETV5*.

To explore the functional implications of these changes, we performed GO enrichment analysis on the target genes of each of the 90 cardiac-specific TF regulons. Enrichment in four major functional categories was observed: cardiac conduction and signalling, cardiac morphogenesis and septation, extracellular matrix and tissue organization, and cardiac muscle development and differentiation (Fig. 3c). Pathways related to cardiac muscle development and differentiation showed the most extensive connections.

### Characterisation of hiPSC-CMs Proliferation

**Fig. 4:**
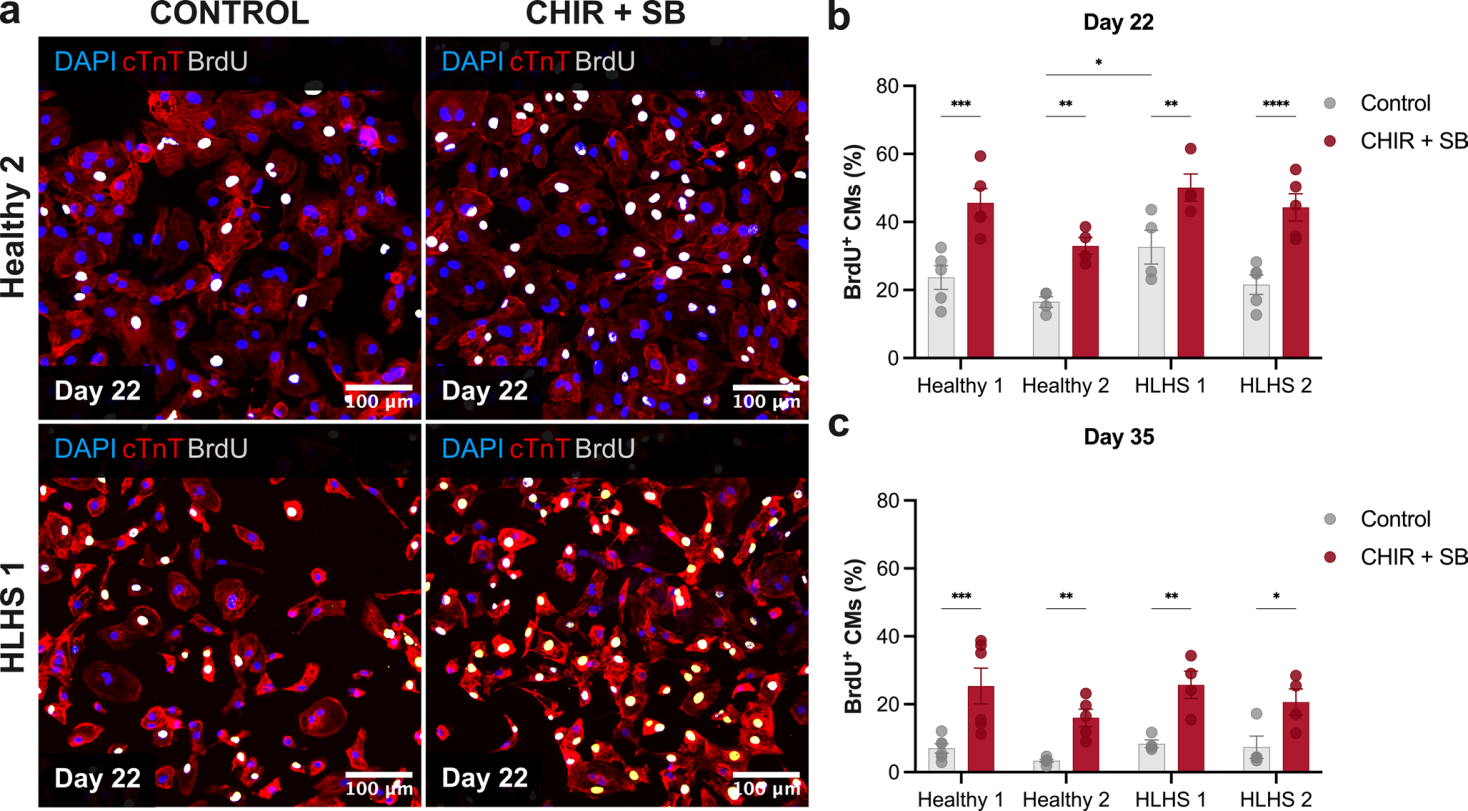
Proliferation of healthy and HLHS cardiomyocytes in basal and pro-mitotic conditions. Proliferation of human induced pluripotent stem cell-derived cardiomyocytes was assessed by bromodeoxyuridine (BrdU) pulse labelling at Day 22 **(a-b**) and Day 35 **(c).** The CMs were treated with either DMSO (control) or a combination of CHIR99021 [5 µM] and SB203580 [10 µM] (CHIR + SB) in the presence of 10 µM BrdU for 24 h. Cells were then fixed and stained for DNA (DAPI, blue), cardiac troponin T (cTnT, red) and BrdU (white). **(a)** Representative immunofluorescence images of control (Healthy 2) and HLHS 1 cardiomyocytes, with or without CHIR + SB treatment. **(b)** The percentage of BrdU-positive cells based on the average intensity of BrdU staining in the nuclei of cTnT+ cardiomyocytes at Day 22. **(c)** The percentage of BrdU-positive cells based on the average intensity of BrdU staining in the nuclei of cTnT-positive cardiomyocytes at Day 35. Data are presented as mean ± SEM, with individual points representing biological replicates from distinct differentiations (*n* = 4–6). Statistical significance was determined using two-way ANOVA followed by Tukey’s post hoc multiple-comparison test. Significance levels are indicated as *p < 0.05, **p < 0.01,***p < 0.001 and, ****p < 0.0001

To explore basal proliferation and the response to mitogenic stimulation, cardiomyocytes were treated with CHIR and SB. Representative immunofluorescence images from Healthy 2 and HLHS 1 at Day 22 illustrate increased BrdU incorporation following CHIR + SB treatment, along with elevated baseline levels in HLHS 1 (Fig. 4a). This pattern was supported by quantification, which showed that under control conditions, HLHS 1 exhibited a significantly higher percentage of BrdU^+^ cardiomyocytes compared to Healthy 2 (1.9-fold increase; *p* < 0.05), indicating an intrinsically elevated proliferative capacity (Fig. 4b). Upon CHIR + SB stimulation at Day 22, all cell lines exhibited significant increases in BrdU^+^ cardiomyocytes compared to their respective controls: Healthy 1 (1.6-fold, *p* < 0.001), Healthy 2 (1.9-fold, *p* < 0.01), HLHS 1 (1.5-fold, *p* < 0.01), and HLHS 2 (1.8-fold, *p* < 0.0001) (Fig. 4b). In contrast, baseline BrdU incorporation at Day 35 was comparable across all cell lines (Fig. 4c). CHIR + SB stimulation at this later time point elicited robust proliferative responses in all lines: Healthy 1 (3.6-fold, *p* < 0.001), Healthy 2 (4.8-fold, *p* < 0.01), HLHS 1 (3.5-fold, *p* < 0.01), and HLHS 2 (2.8-fold, *p* < 0.05) (Fig. 4c). For each time point, we performed a fold-change analysis of BrdU⁺ cardiomyocytes induced by CHIR + SB relative to the corresponding control condition, and quantified average BrdU and cTnT intensities. Results for Day 22 are shown in Additional file 1: Fig. S4a–c, and for Day 35 in Additional file 1: Fig. S4d–f.

### Characterisation of Hypertrophic Responses

#### Basal and ET-1-induced Stress Responses in hiPSC-CMs

**Fig. 5:**
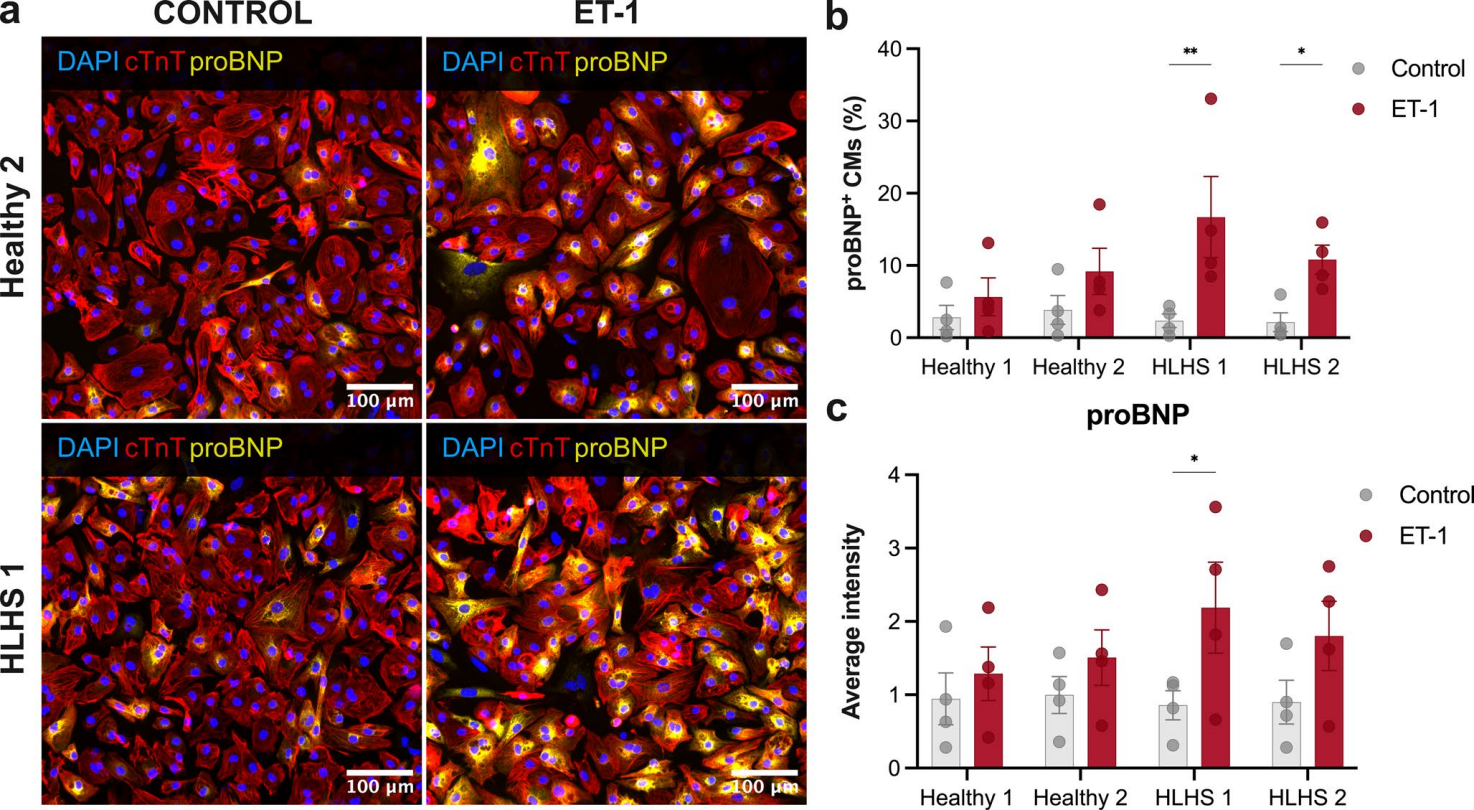
Effects of endothelin-1 (ET-1) on pro-B-type natriuretic peptide (proBNP) expression in healthy and HLHS cardiomyocytes. Human induced pluripotent stem cell-derived cardiomyocytes were treated with either DMSO + BSA (control) or 100 nM ET-1 for 24 h, then fixed and stained for DNA (DAPI, blue), cardiac troponin T (cTnT, red), and proBNP (yellow). **(a)** Representative immunofluorescence images of Healthy 2 and HLHS 1 cardiomyocytes, with or without ET-1 treatment, acquired using a 20× S Plan Fluor ELWD objective. **(b)** The percentage of proBNP-positive cardiomyocytes based on the average intensity of proBNP staining in the perinuclear region of cTnT+ cardiomyocytes. **(c)** Average perinuclear proBNP intensity normalised to Healthy 2. **(b-c)** Data are presented as mean ± SEM, with individual points representing biological replicates from distinct differentiations (*n* = 4). Statistical significance was determined using two-way ANOVA followed by Tukey’s post hoc multiple-comparison test. Results are expressed as mean ± SEM. Significance levels are indicated as *p < 0.05, **p < 0.01

To investigate potential intrinsic differences in hypertrophic stress response between healthy and HLHS cardiomyocytes, we assessed the expression of proBNP, a key marker of cardiac stress, under both basal and ET-1-stimulated conditions. Representative immunofluorescence images demonstrated an increased number of proBNP^+^ cardiomyocytes in HLHS 1 following ET-1 treatment, while Healthy 2 cardiomyocytes exhibited a detectable but non-significant increase (Fig. 5a). This observation was supported by quantitative analysis, which revealed significant increases in the number of proBNP^+^ cardiomyocytes in HLHS 1 and HLHS 2 (7.1-fold, *p* < 0.01 and 5-fold, *p* < 0.05 over control, respectively), whereas Healthy 1 and Healthy 2 showed non-significant increases (2-fold and 2.4-fold, respectively; Fig. 5b). A similar trend was observed in the quantification of perinuclear proBNP intensity (Fig. 5c), and cTnT expression remained consistent across all conditions (Additional file 1: Fig. S5).

#### Cyclic Mechanical Stretching

**Fig. 6:**
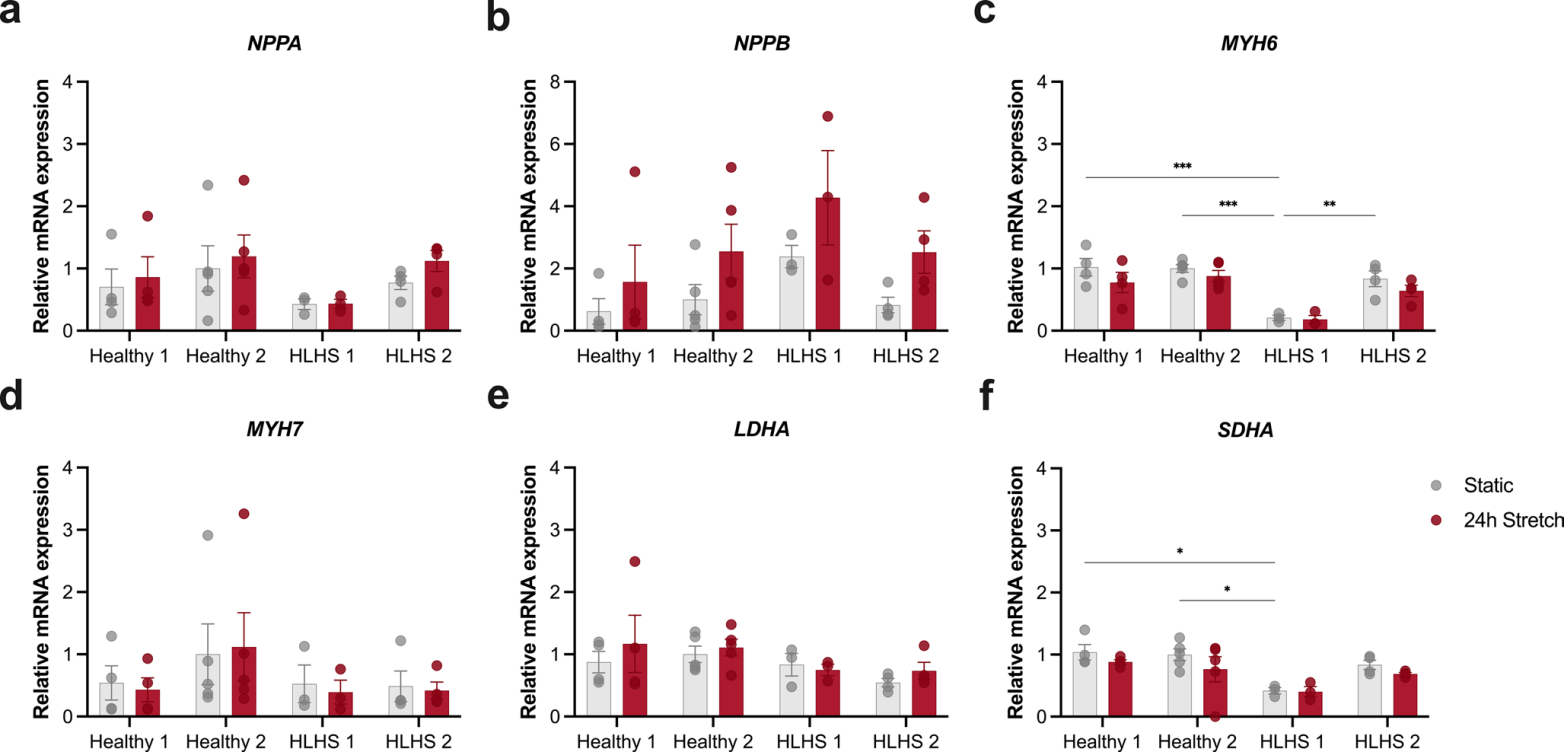
Effects of cyclic mechanical stretching on stretch-responsive gene expression in healthy and HLHS cardiomyocytes. Human pluripotent stem cell-derived cardiomyocytes were subjected to 24 h of cyclic mechanical stretch, whereafter mRNA expression was measured by qPCR. The analysed genes represent multiple aspects of the cardiomyocyte response to mechanical stress, including canonical hypertrophic markers (*NPPA*, *NPPB*), sarcomeric isoform regulation (*MYH6*, *MYH7*), and metabolic enzymes involved in cellular energy metabolism (*LDHA*, *SDHA*). **(a)** Relative mRNA expression of *NPPA *(natriuretic peptide A). **(b)** Relative mRNA expression of *NPPB *(natriuretic peptide B). **(c)** Relative mRNA expression of *MYH6* (Myosin Heavy Chain 6). **(d)** Relative mRNA expression of *MYH7* (Myosin Heavy Chain 7). **(e)** Relative mRNA expression of *LDHA*. **(f)** Relative mRNA expression of *SDHA* (Succinate dehydrogenase complex subunit A). Data are normalised to the average of Healthy 2 control and presented as mean ± SEM, with individual points representing biological replicates from distinct differentiations (*n *= 3–5). Statistical significance was determined using two-way ANOVA followed by Tukey’s post hoc multiple-comparison test. Results are expressed as mean ± SEM. Significance levels are indicated as *p < 0.05, **p < 0.01, and ***p < 0.001

Cardiomyocytes are constantly exposed to mechanical forces in vivo, and their ability to adapt is critical for maintaining heart function. To investigate mechanotransduction differences between healthy and HLHS cardiomyocytes, we applied cyclic stretch for 24 and 48 h and assessed stretch-induced transcriptional responses, including hypertrophic signalling, contractility remodelling, and metabolic regulation. We first validated our stretch model by assessing two canonical stretch-responsive hypertrophic markers, *NPPA* and *NPPB*. Under static conditions, their expression levels were comparable between HLHS and healthy cardiomyocytes. Following 24 h of cyclic stretch, both groups showed a non-significant trend toward increased *NPPA* and *NPPB* expression (Fig. 6a and b). Given that mechanical stress can also affect contractile gene regulation and myosin isoform balance, we additionally measured the expression of MYH6 and MYH7. Under static conditions, MYH6 expression in HLHS 1 was ~ 20% of Healthy 1 (*p* < 0.001), ~ 21% of Healthy 2 (*p* < 0.001), and ~ 25% of HLHS 2 (*p* < 0.01; Fig. 6c), suggesting intrinsic differences in contractile gene expression. After 24 h of stretch, *MYH6* showed a downward trend in both healthy and HLHS cardiomyocytes. *MYH7* expression (Fig. 6d) and *MYH6/MYH7* ratio (Additional file 1: Fig. S6) remained stable across all conditions, indicating that short-term mechanical stimulation did not alter myosin isoform mRNA expression. To assess potential metabolic adaptations, we evaluated expression of LDHA and SDHA, key enzymes in glycolysis and oxidative phosphorylation, respectively. LDHA expression did not differ among groups and remained unchanged following 24-h stretch (Fig. 6e). By contrast, SDHA expression was lower in HLHS 1 under static conditions (~ 40% of Healthy 1, *p* < 0.05; and ~ 42% of Healthy 2, *p* < 0.05) and was not affected by mechanical stimulation (Fig. 6f). We also examined cardiac transcription factors *NKX2.5* [7], *MEF2C* [53, 54], *HES1* [55] and *CSRP3* [51], which regulate cardiomyocyte development and stress responses. No notable changes were observed under static or stretched conditions (Additional file 1: Fig. S6). The results of the 48-hour stretching experiments mirrored those observed at 24 h, suggesting that gene expression differences between HLHS and healthy cardiomyocytes are stable and not substantially altered by prolonged mechanical stimulation (Additional file 1: Fig. S7). Thus, it seems that mechanical stimulation of hiPSC-CMs has less effect on cellular function compared with ET-1 stimulation.

## Discussion

The results of this study indicate that patient-derived HLHS cardiomyocytes exhibit heightened vulnerability to stress, as demonstrated through both transcriptomic profiling of unstimulated cells and functional stress response assays. These findings suggest impaired adaptive mechanisms, potentially contributing to reduced cardiac resilience in HLHS. Maternal metabolic disease [20–25] and maternal hypertension [56] are well known risk factors for CHD in the offspring, and both conditions are likely to result in exposure to increased metabolic and oxidative stress in the developing embryo and foetus. Thus, genetic predisposition for reduced tolerance to environmental stressors in the developing heart may contribute to the multifactorial aetiology of CHD.

Transcriptomic profiling of HLHS cardiomyocytes indicated downregulation of genes involved in metabolic resilience and antioxidant defence, such as *ENO1* and *SESN2*, wherein *ENO1* plays a crucial role in maintaining glycolytic flux under hypoxic conditions, facilitating ATP production when oxidative phosphorylation is compromised, thus having a protective role during cardiac stress [57]. Interestingly, downregulation of *ENO1* has also been observed in a left ventricle cardioid model harbouring the transcription factor *FOXF1* knockout recapitulating CHD [58]. *SESN2* is integral to regulating oxidative stress responses and maintaining metabolic homeostasis; its deficiency has been linked to impaired cardiac protection and increased susceptibility to oxidative damage [59, 60]. *SESN2* polymorphism has also been associated with CHD [61]. Additional reductions in *APOA1* and *SOD3*, which are involved in lipid regulation and reactive oxygen species detoxification respectively, further suggest compromised oxidative stress defence mechanisms. These signatures are consistent with prior reports of mitochondrial dysfunction and apoptosis in HLHS hiPSC-CMs under metabolic challenge [62, 63], and may reflect pathophysiologic mechanisms behind maternal metabolic disease as a risk factor for CHD in the offspring [20–25].

While differential gene expression analysis identified specific genes with altered expression, we sought to determine whether these changes reflected coordinated disruption of transcriptional regulatory networks. Regulon analysis revealed widespread disruption of transcriptional regulatory networks in HLHS cardiomyocytes. Of 645 significantly differentially active regulons, the vast majority showed decreased activity in HLHS compared to healthy controls, with a similar pattern among cardiac-specific transcription factors. While the magnitude of these changes was modest, even small alterations in transcription factor activity can have amplified downstream effects on target gene expression networks [64]. The affected regulons span multiple aspects of cardiac function, including cardiac muscle development, morphogenesis, conduction, and extracellular matrix organization. This pattern of coordinated transcriptional downregulation may suggest impairments in differentiation, function, and interaction with other cell types, potentially leading to systemic regulatory dysfunction rather than isolated pathway defects. This may affect proper cardiac development and stress adaptation.

To further probe cardiomyocyte adaptability, we examined their responses to external stressors, including endothelin-1 (ET-1) and cyclic mechanical stretch. The HLHS lines showed significantly greater increases in proBNP expression during ET-1 stimulation compared to controls, indicating a heightened sensitivity to pro-hypertrophic hormonal challenge. Indeed, ET-1 stimulation may recapitulate increased stress in the developing heart well, as several lines of evidence suggest that elevated plasma ET-1 is one of the mediators of vascular complications in individuals with metabolic disease and it is an important mediator in uteroplacental circulation and foetal vascular function [65] and [66]. ET-1 is also known to play an important role in regulating cardiomyocyte differentiation during heart development [66]. The heightened responses to ET-1 stimulation in HLHS hiPSC-CMs may thus reflect disease-specific vulnerability to external stressors such as maternal metabolic disease.

In contrast, responses to mechanical stretch were more heterogeneous. Cyclic stretch did not substantially alter gene expression profiles in a manner that consistently separated HLHS from controls. A previous study applying stretch to HLHS-CMs observed downregulation of cell cycle genes and upregulation of structural genes [63], partially similar to our findings, although the overall transcriptional response in our dataset was modest. Overall, these results point to transcriptional heterogeneity among HLHS lines in their response to biomechanical cues, underscoring the complexity of modelling this condition and the value of patient-specific approaches.

Previous studies have consistently reported reduced basal proliferation in HLHS cardiomyocytes compared to controls, including in HLHS tissue [67, 68], iPSC models [69], and animal models [69–71]. To assess both basal and inducible proliferative capacity, we used CHIR99021 and SB203580, which act synergistically to enhance cardiomyocyte proliferation through complementary mechanisms [47]. In our study, HLHS cardiomyocytes showed either similar or increased proliferation at baseline compared to controls, challenging the idea of a uniformly reduced proliferative capacity. A recent study using CHIR99021 to stimulate proliferation reported lower proliferation in HLHS cells relative to controls at both baseline and after treatment [63]. Importantly, their data, while focused on intergroup comparison, also showed that HLHS cardiomyocytes were capable of increasing proliferation in response to CHIR. This is consistent with our findings, which demonstrate that HLHS cardiomyocytes retain mitogenic responsiveness despite baseline variability.

In addition to metabolic and proliferative abnormalities, HLHS cardiomyocytes exhibited changes in the expression of several ion channels. Transcriptomic signatures showed reduced expression of key pacemaker channel genes, including *HCN2* and *HCN4*, which are known to be involved in sinoatrial node function and the regulation of cardiac automaticity. Notably, genetic variants in *HCN4* have been implicated in atrial arrhythmias, atrioventricular nodal disease, and left ventricular noncompaction [72]. In parallel, expression of potassium channel genes *KCNA5* and *KCNE1*, which have been associated with atrial fibrillation and cardiac repolarization anomalies, respectively [73–75], was also reduced in HLHS cardiomyocytes. These changes were accompanied by decreased expression of *SHOX2*, a transcription factor implicated in sinoatrial node development and atrial rhythm regulation [76–78]. While the direct functional consequences of these transcriptomic changes were not assessed in this study, such alterations may reflect an intrinsic molecular predisposition affecting pathways related to cardiac electrical regulation in congenital heart disease patients.

These findings provide insight into how HLHS cardiomyocytes integrate, or fail to integrate, key developmental cues. While maternal conditions such as diabetes and hypertension are known to elevate circulating ET-1, our data show that HLHS cardiomyocytes exhibit a markedly heightened response to ET-1. This suggests that HLHS cells may be primed for pathological activation when challenged by hormonal cues during development. Taken together, these results emphasize the importance of gene–environment interactions and support a model in which inappropriate responses to developmental stressors contribute to disease progression. This insight may help explain the clinical variability observed in HLHS and highlights the value of patient-specific models for therapeutic development.

### Study Limitations and Future Directions

While our study provides valuable insights into the cellular and molecular basis of HLHS, the small number of patient-derived cell lines limits our ability to fully capture the spectrum of HLHS heterogeneity. The variable responses observed among our HLHS lines reflect the complex, patient-specific nature of this disorder and suggest that multiple pathogenic mechanisms may exist. Nevertheless, despite the limited sample size, our findings identify common maladaptive processes in HLHS cardiomyocytes, supporting the validity and broader relevance of our conclusions.

Another limitation is that our hiPSC-CMs were studied in 2D monoculture systems, lacking the native cardiac microenvironment and heterotypic cell-cell interactions that could influence cellular behaviour, indicating that additional non-myocyte-derived pathways and mechanisms not identified here are likely to contribute to the disease development. While this simplification may exclude important contributions from non-myocyte-derived pathways, it allowed us to focus specifically on intrinsic cardiomyocyte defects. Moreover, the relative immaturity of hiPSC-CMs, often considered a limitation [79], may actually serve as a strength in this context, as it mirrors the developmental stage during which congenital heart defects such as HLHS originate.

Future studies should expand the cohort of patient-derived lines to more robustly characterise cellular heterogeneity and patient-specific variability. Increasing the number of lines would help validate and refine the molecular and cellular mechanisms identified here. In addition, employing advanced culture systems, such as cardiac co-culture models or engineered heart tissues, could better replicate the in vivo cardiac microenvironment and provide further insights into the multicellular and biomechanical interactions underlying HLHS. Despite the inherent complexity and heterogeneity of HLHS, these advanced patient-derived in vitro models are expected to unravel cellular and molecular mechanisms underlying HLHS, which may lead to improved diagnostic and preventative and/or therapeutic strategies in the future.

## Conclusion

Our results indicate that patient-derived HLHS cardiomyocytes display impaired function and heightened sensitivity to oxidative and metabolic challenges. Despite the heterogeneity among patient lines, these common features suggest shared vulnerability mechanisms in HLHS. Collectively, these findings support the view that congenital heart defects, including HLHS, may arise from a complex interplay between genetic predisposition and adverse developmental conditions during cardiac development.

## Supplementary Information

Below is the link to the electronic supplementary material.

Supplementary Material 1

## Data Availability

The RNAseq data are freely available in the Gene Expression Omnibus (GEO) repository (accession number GSE194103). All other datasets generated during and analysed during the current study are available from the corresponding author on reasonable request.
