# Supplementary material for: Hypoplastic Left Heart Syndrome Cardiomyocytes Exhibit Intrinsic Stress Vulnerabilities and Augmented Stress Responses in vitro
Source: Stem Cell Rev Rep. 2026 May 6;22(5):2549–67. doi: 10.1007/s12015-026-11127-3 (PMC13241447; doi:10.1007/s12015-026-11127-3)
Supplement: Supplementary file 1 — Supplementary Material 1 [file 12015_2026_11127_MOESM1_ESM.pdf]

Supplementary information

Hypoplastic left heart syndrome cardiomyocytes exhibit intrinsic stress vulnerabilities and augmented stress responses *in vitro*

Varela M[1,2*], Ampuja M[2], Broberg M[2,3], Ramste A[2], Talman V[1,4*†], Helle E[2,5*†]

[1]Drug Research Program, Division of Pharmacology and Pharmacotherapy, Faculty of Pharmacy, University of Helsinki, Helsinki, Finland

[2]Stem Cells and Metabolism Research Program (STEMM), Research Programs Unit, Faculty of Medicine, University of Helsinki, Helsinki, Finland

[3]Institute for Molecular Medicine Finland (FIMM), HiLIFE, University of Helsinki, Helsinki, Finland

[4]Research Centre for Integrative Physiology and Pharmacology, Institute of Biomedicine, Faculty of Medicine, University of Turku, Turku, Finland

[5]New Children's Hospital, Paediatric Research Centre, Helsinki University Hospital, Helsinki, Finland

* co-senior authors

[†]Corresponding authors:

Virpi Talman, Faculty of Medicine, University of Turku, Kiinamyllynkatu 10, 20520 Turku, Finland. Email: virpi.talman@utu.fi

Emmi Helle, Faculty of Medicine, University of Helsinki, Haartmaninkatu 8, Helsinki, Finland. Email: emmi.helle@helsinki.fi

Table of Contents

Supplementary Tables S1-S2 .... p. 2

Supplementary Figures S1-S6 .... pp. 3-8

Supplementary Tables

Supplementary Table S1. Clinical and Genetic Characteristics of HLHS cell lines

Study ID	Source line ID	Sex	Genetic variant	Cardiac phenotype
HLHS 1	HEL 149	male	ERBB2 (chr17:37873630C>T, p.Arg599Cys, rs369903296; hg19) [1]	HLHS, COA, HAA, LSVC
HLHS 2	HEL 218	male	-	HLHS, AA, MS
HLHS 3	HEL 169	male	NOTCH1 (chr9:139404414C>T, c.2741-1G>A; predicted loss-of-function)	HLHS
HLHS 4	HEL 216	male	-	BAV, COA, HAA, ASD

HLHS = hypoplastic left heart syndrome, BAV = bicuspid aortic valve, COA = coarctation of the aorta, HAA = hypoplastic aortic arch, ASD = atrial septal defect, LSVC = left superior vena cava, AA =aortic atresia and MS = mitral stenosis.

References:

1. Ampuja M, Ericsson S, Paatero I, Chowdhury I, Villman J, Broberg M, et al. The ERBB2 c.1795C>T, p.Arg599Cys variant is associated with left ventricular outflow tract obstruction defects in humans. Human Genetics and Genomics Advances. 2025;6:100446. https://doi.org/10.1016/j.xhgg.2025.100446

Supplementary Table S2. Commercial TaqMan® Gene Expression Assays.

Gene Symbol	Gene Name	Assay ID
18S	rRNA, eukaryotic 18S ribosomal RNA	4352930E
ACTB	Beta-actin	4352935E
CSPR3	Cysteine and glycine rich protein 3	Hs00185787_m1
HES-1	Hes family bHLH transcription factor 1	Hs00172878_m1
LDHA	Lactate dehydrogenase A	Hs01378790_g1
MEF2C,	Myocyte enhancer factor 2C	Hs00231149_m1
MYH6	Myosin heavy chain 6	Hs01101425_m1
MYH7	Myosin heavy chain 7	Hs01110632_m1
NKX2-5	NK2 homeobox 5	Hs00231763_m1
NPPA	Atrial natriuretic peptide	Hs00383230_g1
NPPB	B-type natriuretic peptide	Hs01057466_g1
SDHA	Succinate dehydrogenase complex flavoprotein subunit A	Hs07291714_m1

Supplementary Figures

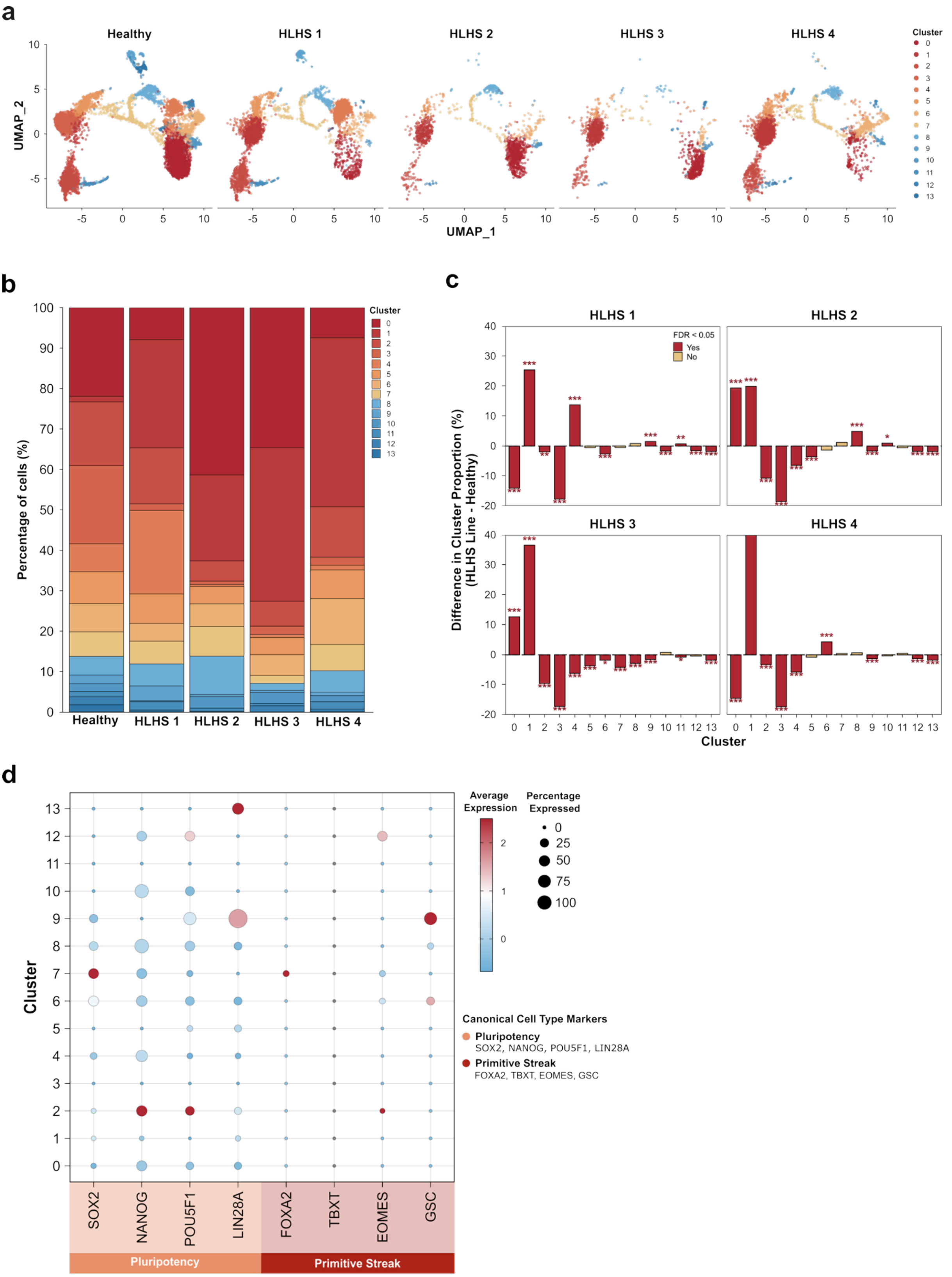

Supplementary Figure S1. Single-cell characterization and cluster composition across individual HLHS patient lines compared to healthy control. **(a)** UMAP visualization of iPSC-derived cardiomyocytes split by sample, showing cluster distribution (0-13) across the pooled healthy control and four individual HLHS patient lines (HLHS 1-4). Each point represents a single cell, colored by cluster identity. **(b)** Stacked bar chart showing the percentage distribution of cells across 14 clusters for each sample. Bars represent the proportion of cells in each cluster (0-13) as a percentage of total cells per sample. **(c)** Comparison of cluster proportions between each individual HLHS line and the healthy control. Bar charts show the difference in cluster proportion (HLHS 1-4 - Healthy) for each cluster. Red bars indicate clusters with significant differences (FDR < 0.05), while yellow bars indicate non-significant differences. Asterisks denote significance levels: *P < 0.05, **P < 0.01, ***P < 0.001. Positive values indicate enrichment in HLHS; negative values indicate depletion compared to healthy control. **(d)** Expression of canonical cell type markers across clusters. Dot plot displays average expression (color intensity, blue to red) and percentage of cells expressing (dot size) pluripotency markers (SOX2, NANOG, POU5F1, LIN28A) and primitive streak markers (FOXA2, TBXT, EOMES, GSC) across all 14 clusters (0-13). Markers are grouped by cell type identity as indicated by the colored bar below the x-axis.

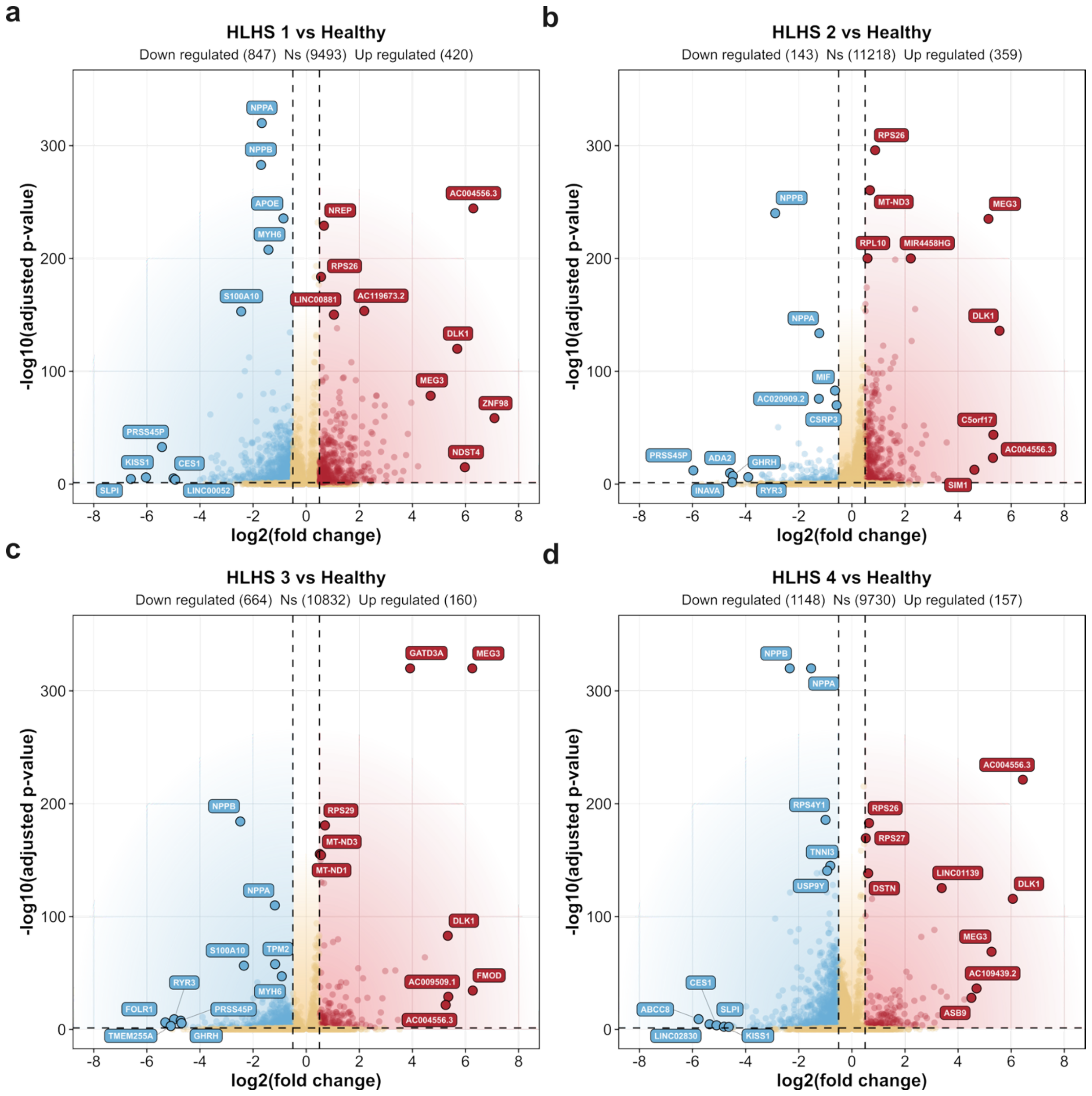

Supplementary Figure S2. Differential gene expression and functional enrichment in HLHS. (a) Volcano plot showing differentially expressed genes between HLHS 1 and healthy samples. **(b)** Volcano plot showing differentially expressed genes between HLHS 2 and healthy samples. **(c)** Volcano plot showing differentially expressed genes between HLHS 3 and healthy samples. **(d)** Volcano plot showing differentially expressed genes between HLHS 4 and healthy samples. Blue dots represent downregulated genes, red dots represent upregulated genes, and yellow dots represent genes that were not differentially expressed. The top 5 genes with the most signficant adjusted p-value and top 5 genes with the highest log2(fold change) are labeled. Vertical dashed lines indicate log2(fold change) thresholds of -0.5 and 0.5; horizontal dashed line indicates adjusted p-value threshold of 0.05.

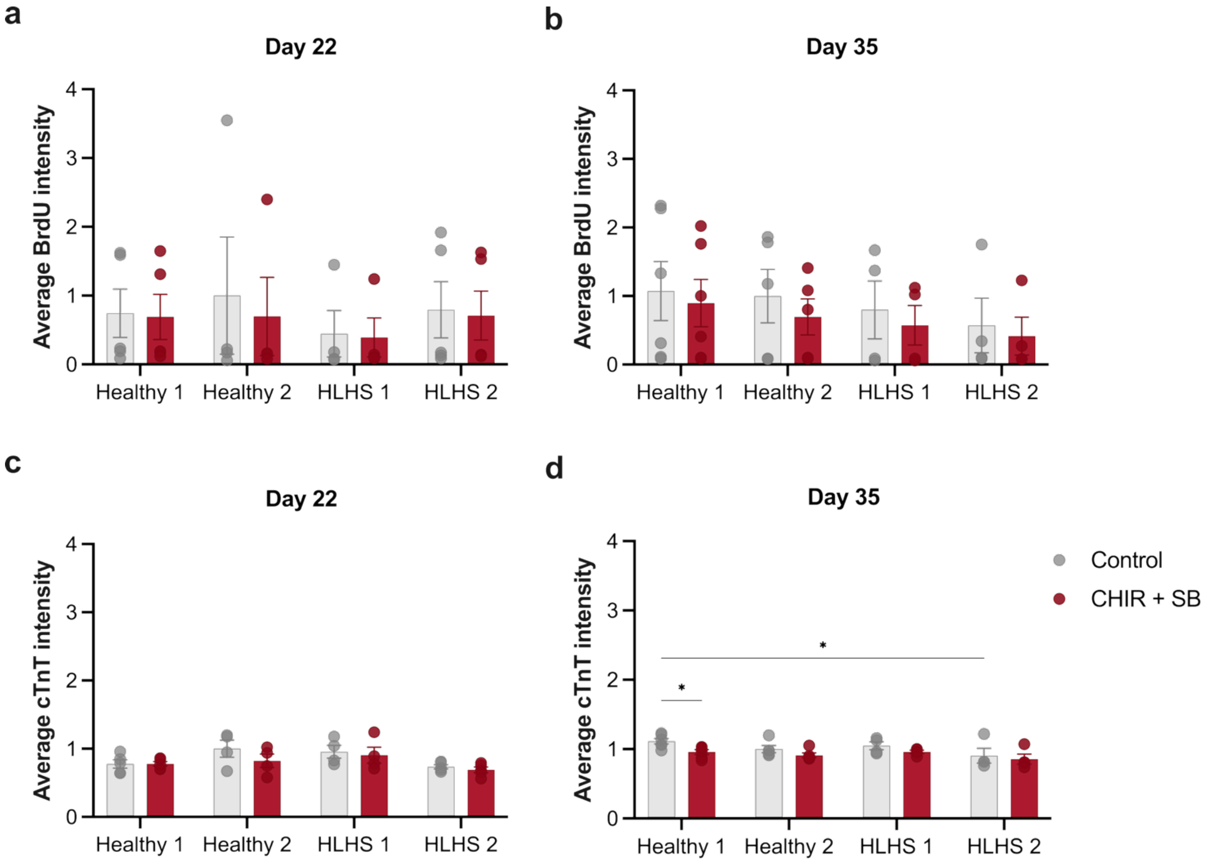

Supplementary Figure S3: Quantification of BrdU and cTnT intensity in healthy and HLHS samples. (a-b) Quantification of average BrdU intensity at Day 22 **(a)** and Day 35 **(b). (c-d)** Quantification of average cTnT intensity at Day 22 **(c)** and Day 35 **(d)**. Data are presented as mean ± SEM, with individual points representing biological replicates from distinct differentiations (n = 4-6). Statistical significance was determined using two-way ANOVA followed by Tukey's post hoc multiple-comparison test. Significance levels are indicated as *p < 0.05.

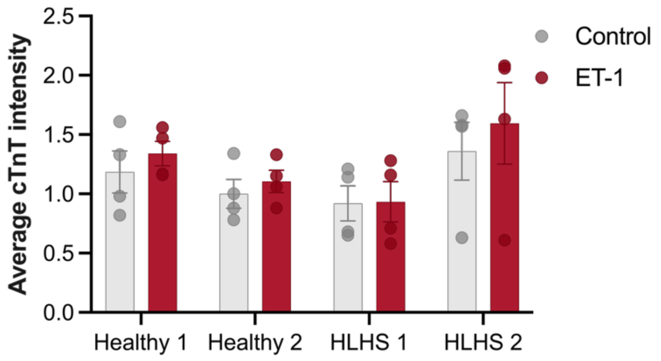

Supplementary Figure S4: Quantification of cTnT intensity in healthy and HLHS samples. Data are presented as mean ± SEM, with individual points representing biological replicates from distinct differentiations (n = 4). Statistical significance was determined using two-way ANOVA followed by Tukey's post hoc multiple-comparison test.

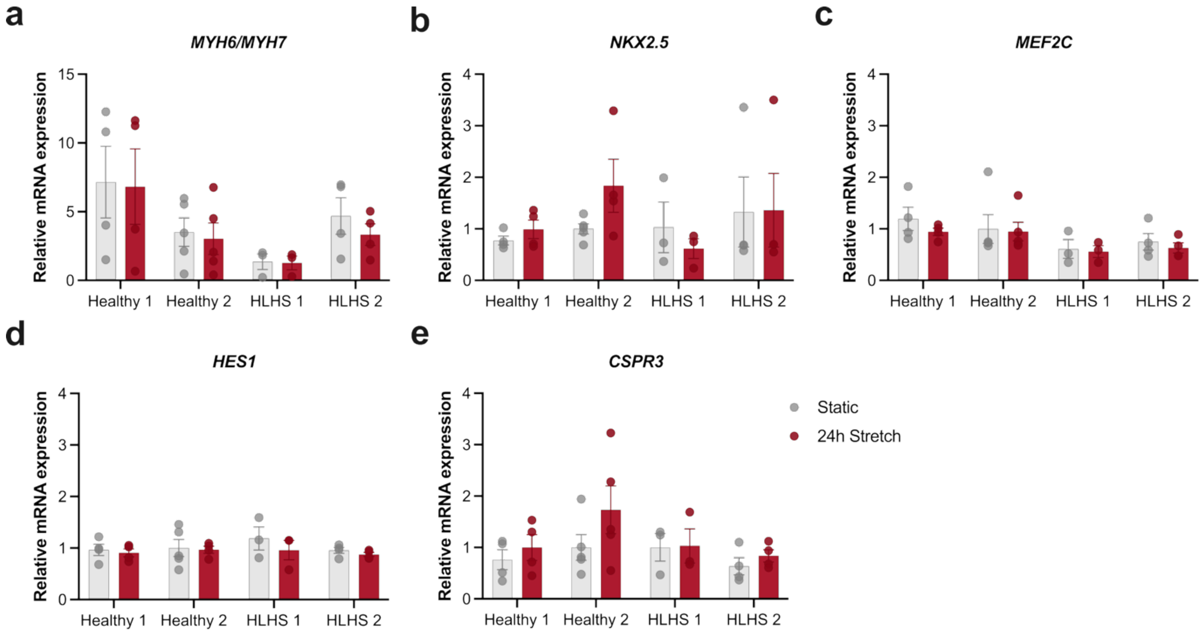

Supplementary Figure S5. Effects of 24h cyclic mechanical stretching on hypertrophic gene expression in healthy and HLHS cardiomyocytes. Human pluripotent stem cell-derived cardiomyocytes were subjected to 24h of cyclic mechanical stretch (10–21%, 0.5 Hz, whereafter mRNA expression was measured by qPCR. **(a)** Relative mRNA expression of the MYH6/MYH7 (myosin heavy chain 6/7) ratio. **(b)** Relative mRNA expression of NKX2.5 (NK2 homeobox 5). **(c)** Relative mRNA expression of MEF2C (myocyte enhancer factor 2C). **(d)** Relative mRNA expression of HES1 (hes family bHLH transcription factor 1). **(e)** Relative mRNA expression of CSRP3 (cysteine and glycine-rich protein 3). Data are normalised to the average of Healthy 2 control and presented as mean ± SEM, with individual points representing biological replicates from distinct differentiations (n = 3-5). Statistical significance was determined using two-way ANOVA followed by Tukey's post hoc multiple-comparison test.

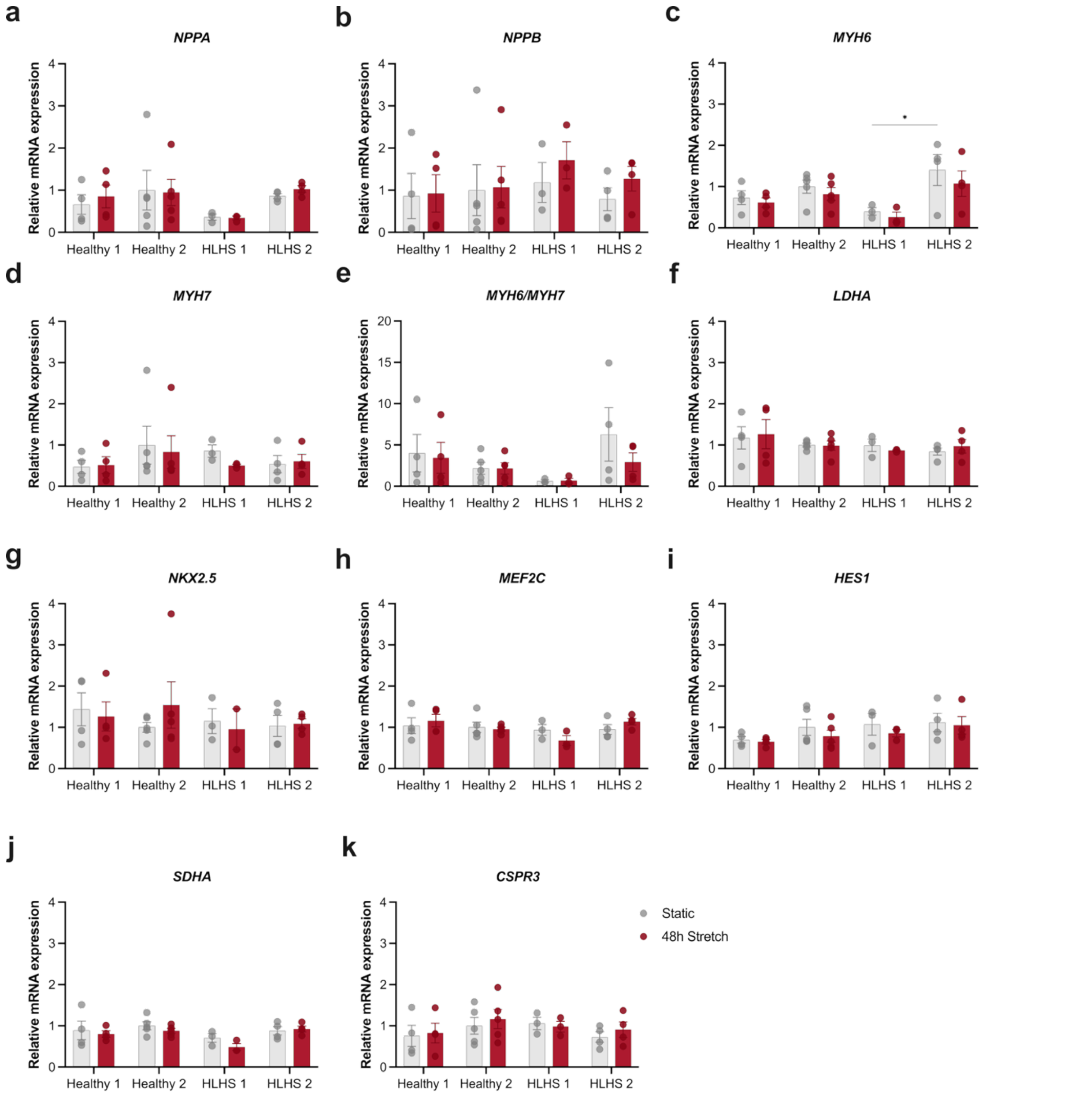

Supplementary Figure S6. Effects of 48h cyclic mechanical stretching on hypertrophic gene expression in healthy and HLHS cardiomyocytes. Human pluripotent stem cell-derived cardiomyocytes were subjected to 48h of cyclic mechanical stretch (10–21%, 0.5 Hz, whereafter mRNA expression was measured by qPCR. **(a)** Relative mRNA expression NPPA (natriuretic peptide A). **(b)** Relative mRNA expression of NPPB (natriuretic peptide B). **(c)** Relative mRNA expression of MYH6 (Myosin Heavy Chain 6). **(d)** Relative mRNA expression of MYH7(Myosin Heavy Chain 7). **(e)** Relative mRNA expression of MYH6/MYH7. **(f)** Relative mRNA expression of LDHA. **(g)** Relative mRNA expression of NKX2.5 (NK2 homeobox 5). **(h)** Relative mRNA expression of MEF2C (myocyte enhancer factor 2C). **(i)** Relative mRNA expression of HES1 (hes family bHLH transcription factor 1). **(j)** Relative mRNA expression of SDHA (succinate dehydrogenase complex flavoprotein subunit A). **(k)** Relative mRNA expression of CSPR3 (cysteine and serine-rich protein 3). Data are normalised to the average of Healthy 2 control and presented as mean ± SEM, with individual points representing biological replicates from distinct differentiations (n = 2-5). Statistical significance was determined using two-way ANOVA followed by Tukey's post hoc multiple-comparison test. Significance levels are indicated as *$p < 0.05$.
